# *Flos Puerariae*-*Semen Hoveniae* medicinal pair extract ameliorates DSS-induced inflammatory bowel disease through regulating MAPK signaling and modulating gut microbiota composition

**DOI:** 10.3389/fphar.2022.1034031

**Published:** 2022-12-07

**Authors:** Xiaofan Chen, Jiahui Zhang, Rui Li, Hua Zhang, Yong Sun, Li Jiang, Xiaoya Wang, Yaokun Xiong

**Affiliations:** ^1^ College of Pharmacy, Jiangxi University of Chinese Medicine, Nanchang, China; ^2^ Evidence-Based Medicine Research Centre, Jiangxi University of Chinese Medicine, Nanchang, China; ^3^ State Key Laboratory of Food Science and Technology, Nanchang University, Nanchang, China

**Keywords:** *Flos Puerariae and Semen Hoveniae*, antioxidant, anti-inflammation, gut microbiota, UPLC-LTQ-orbitrap-MS/MS

## Abstract

**Background:** Inflammatory bowel disease (IBD) is a global gastrointestinal disease characterized by relapsing and remitting inflammatory conditions. *Flos Puerariae* (the flower of *Pueraria lobata* [Willd.] Ohwi and *P. thomsonii* Benth.) and *Hovenia dulcis* Thunb. (Rhamnaceae) are traditional Chinese medicines. This medicinal pair has been used to treat various diseases due to its excellent anti-oxidant and anti-inflammatory activity. However, the effects of extracts from these plants on dextran sulfate sodium (DSS)-induced colitis have not been investigated; further study is needed to improve the understanding of their mechanisms of action and potential applications.

**Methods:** The chemical constitution of extracts from *Flos Puerariae* and *Semen Hoveniae* (PHE) was analyzed using UPLC-LTQ-Orbitrap-MS/MS. The protective effects of PHE on mice with DSS-induced colitis were evaluated through assessment of body weight loss, disease activity index (DAI) score, colon length shortening, and pathological changes. The levels of inflammatory cytokines were determined by ELISA and RT-qPCR. Biomarkers of oxidative stress (ROS, CAT, SOD, MDA, and T-AOC) were analyzed using biochemical kits. The expression of MAPK proteins was determined by Western blotting analysis. Gut microbiota were analyzed *via* 16S rRNA sequencing.

**Results:** Chemical composition analysis indicated that PHE contains various bioactive compounds, including puerarin, kakkalide, tectoridin, and genistin. The findings from this study suggest that PHE could effectively modulate histopathological score, inflammatory cell infiltration, and inflammatory factor secretion. Notably, PHE ameliorated oxidative stress by inhibiting activation of the MAPK pathway, leading to decreased inflammatory mediators and restored antioxidant enzyme activity. Furthermore, PHE treatment regulated the composition of the gut microbiota by increasing the abundance of benign bacteria, such as *Akkermansia*, and reducing the abundance of harmful bacteria, such as *Proteobacteria*.

**Conclusion:** The findings from this study demonstrate the mechanism underlying the amelioration of DSS-induced intestinal oxidative stress by PHE and its positive impact on the restoration of the composition of gut microbiota.

## Introduction

Human inflammatory bowel disease (IBD), characterized by relapsing and remitting inflammatory conditions in the gastrointestinal tract, is becoming a global disease with elevating prevalence. Clinical drugs, such as immune-suppressants and glucocorticoids, used in IBD therapy have unavoidable side effects on the gastrointestinal tract and other organs. Natural-origin bioactive compounds, such as polyphenolic acids and flavonoids, have gained abundant attention for their effectiveness and lack of discernible toxicity in animals. In particular, phytochemicals from traditional Chinese medicine (TCM) display excellent efficacy and safety in the treatment of IBD. For instance, ginseng (*Panax ginseng* C.A.Mey.) extract was shown to exhibit anti-inflammatory activity through inhibition of the MAPK/NF-κB pathway and activation of autophagy and p62-Nrf2-Keap1 signaling in DSS-induced mice (Yang et al., 2022). Extracts from *Atractylodis Rhizoma* (a folk medicine made of the dried rhizome of *Atractylodes lancea* [Thunb.] DC [Aster]) significantly attenuated the symptoms of colitis by reducing the levels of inflammatory cytokines and inhibiting the phosphorylation of proteins in the MAPK and NF-κB signaling pathways (Lin et al., 2022). The beneficial effects of TCM extracts have been closely associated with the presence of specific compounds.

*Flos Puerariae*, the flower of *Pueraria lobata* (Willd.) Ohwi is rich in flavonoids, isoflavones, saponins, sterols, and alkaloids (Wei et al., 2022). Among these, genistein, an isoflavone, has been widely studied and found to protect cells against oxidative stress by reducing pro-inflammatory mediator production (Chiorcea-Paquim et al., 2020). Tectoridin, the main isoflavone of *Flos Puerariae*, can be transformed by intestinal bacteria into tectorigenin, which exhibits more potent activity, including enhanced anti-inflammatory effects and benefits in the treatment of liver injury (Chien et al., 2006). In addition, puerarin, the primary active phytoisoflavone isolated from *Pueraria lobata* and *Flos Puerariae*, has been widely reported to alleviate oxidative stress and repress inflammation (Guan et al., 2020). *Semen Hoveniae* is a matured seed of *Hovenia dulcis*. Recent studies have revealed that *H. dulcis* contain abundant bioactive compounds, such as flavonoids, triterpene saponins, alkaloids, organic acids, and phenylpropanoids (Chul-Yung et al., 2018). Used in China from ancient times through the present day, the *Flos Puerariae* and *Semen Hoveniae* (PH) medicinal pair has been a common treatment for the sustenance of the liver redox balance and prevention of liver disease in the context of alcohol intoxication (Cho et al., 2016). Recent evidence has suggested that PH extracts (PHE) have therapeutic effects on alcoholic liver disease by promoting lipid metabolism, improving antioxidant activity, and resisting lipid peroxidation (Xu et al., 2020).

Studies confirmed that alcohol use exacerbates intestinal inflammation and results in gut dysbiosis (Schnabl and Brenner, 2014). However, the effect of the *Flos Puerariae*-*Semen Hoveniae* medicinal pair on intestinal inflammation and gut dysbiosis remains unclear. In addition, *Flos Puerariae* has been shown to ameliorate the intestinal inflammation of *Drosophila via* modulation of Nrf2/Keap1, JAK-STAT, and Wnt signaling (Yang et al., 2022). Furthermore, *Hoveniae Semen* seu fructus ethanol extract has exhibited anti-inflammatory activity *via* MAPK, AP-1, and STAT signaling in an LPS-stimulated murine macrophage cell line and in peritoneal macrophages from an LPS-stimulated mouse model (Jeong et al., 2019). The aforementioned studies indicate that both *Flos Puerariae* and *Hoveniae Semen* exhibit anti-inflammatory effects *in vitro* and/or *in vivo*.

The gut microbiota, the intestinal epithelium, and the host immune system maintain intestinal homeostasis, while disruption of this balance is associated with the occurrence of intestinal disease. The DSS-induced intestinal dysfunction mouse model represents a condition of relapsing and remitting inflammation in the gastrointestinal tract, which is a hallmark of inflammatory bowel disease (IBD) in humans. In the present study, a DSS-induced mouse model was established to investigate the effect of extracts from *Flos Puerariae–Semen Hoveniae* on gut homeostasis, with analysis of oxidative stress, inflammatory state, and the composition of gut microbiota. In addition, the chemical constitution of PHE was analyzed using a UPLC-LTQ-Orbitrap-MS/MS. This study aimed to characterize the mechanism(s) underlying PHE-mediated mitigation of colitis and other diseases related to intestinal dysfunction.

## Materials and methods

### Materials and chemicals

*Flos Puerariae* and *Semen Hoveniae* were acquired in June from the Jinbei Traditional Chinese Medicine Planting and Breeding Cooperative (Jiangxi, China). DSS reagents were obtained from MP Biomedicals (MP Biomedicals, United States). Enzyme-linked immunosorbent assay (ELISA) kits for IL-1β (lot number E-EL-M0037c), IL-6 (lot number E-EL-M0044c), TNF-α (lot number E-EL-M0049c), and PGE2 (lot number E-EL-M0034c) were obtained from Elabscience (Wuhan, China). Antibodies against PCNA (AH08154078) and CD11b (AB08352478) were purchased from Boster Bio (Pleasanton, United States). Antibodies against inducible nitric oxide synthase (iNOS, 18985-1-AP) and cyclooxygenase-2 (COX-2, 10627-1-BM) were obtained from Abcam (Cambridge, United Kingdom). Antibodies against MAPK pathway proteins were obtained from Cell Signaling Technology (Danvers, MA, United States). HPLC-grade acetonitrile and methanol were obtained from Merck (Darmstadt, Germany). SOD (lot number A001-3-2), MDA (lot number A003-1-2), CAT (lot number A007-1-1), ROS (lot number E004-1-1), and T-AOC (lot number A015-3-1) kits were obtained from Nanjing Jiancheng Bioengineering Institute (Nanjing, China).

### Preparation of samples

PHE was prepared based on the traditional usage of the *Flos Puerariae–Semen Hoveniae* medicinal pair. Briefly, 79.8 g of *Flos Puerariae* was soaked in distilled water for 2 h, followed by decocting extraction for 1.5 h. The process was repeated, and the *Flos Puerariae* extract was obtained by vacuum drying. For *Semen Hoveniae*, an extract was obtained by using 95% ethanol following decoction. The two extracts were mixed at a 5:1 ratio (*Flos Puerariae: Semen Hoveniae*) for the UPLC-MS/MS and animal experiments.

### Chromatography and mass spectrometry

The PHE samples were resolved in distilled water at a concentration of 1 mg/ml for UPLC-MS/MS analysis after filtration through a 0.22-μm membrane. Linear gradient elution was performed according to our previously published method (X. Wang et al., 2020). The mobile phase consisted of water (A) and acetonitrile (B), containing 0.1% formic acid. The separation was achieved using a unitary C18 column (2.1 × 150 mm, 2.8-micron particle size) at 23°C. The flow rate was 0.3 ml/min, and the elution gradient was as follows: 0–1 min, 8% B; 1-18 min, 8%–35% B; 18–19 min, 35%–40% B; 19–28 min, 40%–70% B; 28–36 min, 70%–100% B.

Mass spectrometry was carried out on an Orbitrap mass spectrometer (Thermo Fisher Scientific, CA, United States) equipped with heated electrospray ionization in negative mode. The intensity threshold was set at 1000; the activation type was collision-induced dissociation (CID); other parameters were as follows: heater temperature, 350°C; sheath gas flow rate, 35 arb; aux gas flow rate, 10 arb; sweep gas flow rate, 0 arb; spray voltage, 3.6 kV; capillary temperature, 320°C; mass range collected from *m/z*, 100–1500 Da. The acquired data were analyzed using Xcalibur™ v.2.0 software and Qualbrowser.

### Animal model and drug administration

C57BL/6 mice (male, 7 weeks of age) were housed in an air-conditioned chamber (23 ± 2°C) with a day/night cycle of 12 h. The animal experiments were approved by the Animal Ethics Committee of Jiangxi University of Traditional Chinese Medicine (No. 20210618). All experimental procedures were performed in accordance with ethical requirements. The mice were given free access to drinking water and food for 3 days of acclimation. The mice were then randomly divided into four groups (*n* = 8): a normal control group (NC), a 3% DSS-treated group (DSS), a low-dose PHE-treated group (PHE-L, 185 mg/kg/day), and a high-dose PHE-treated group (PHE-H, 740 mg/kg/day). With the exception of the NC group, the mice were free to drink 3% DSS solution from days 8–14. The mice in the PHE-treated group were gavaged with different doses of PHE from the first to the last day. Body weights and pharmacological responses were recorded daily. To determine the effectiveness of the treatment, the mice were killed and the colon tissues were collected and divided for histological, RT-qPCR, and Western blotting analysis. The disease activity index (DAI) score was evaluated based on body weight loss, stool consistency, rectal bleeding, and mouse condition according to methods used in our previous study (X. Wang et al., 2019).

### Histopathological evaluation

Sections of the colon tissue were fixed in 4% paraformaldehyde for 24 h, embedded in paraffin wax, and then cut using a Leica RM2235 microtome. The sections were then washed and soaked in PBS buffer, stained with hematoxylin–eosin (H&E), and observed *via* light microscopy. An independent team of three investigators, blind to experimental conditions, evaluated the tissue sections and assigned pathological scores based on the integrity of the epithelial structure, the number of crypt cells, the degree of infiltration of inflammatory cells, and the extent of destruction of the mucosal layer, as carried out in our prior study (X. Wang et al., 2019).

### Immunohistochemistry

Briefly, the colon tissue sections were dewaxed in water, and the antigens were retrieved with a citric acid antigen repair solution. The samples were then rinsed three times for 5 min with PBS and blocked with blocking buffer (5% BSA in PBS) at room temperature for 30 min. After rinsing with PBS, the sections were incubated with primary antibodies in a humidified chamber at 4°C overnight. The sections were then rinsed with PBS and incubated with secondary antibodies. After dehydration, the DAB-stained nuclei were re-stained with hematoxylin and the mounted sections were sealed. The nuclei stained with hematoxylin appeared blue, and the expression of CD11b and PCNA was indicated by brownish-yellow staining. The sections were observed using light microscopy.

### Determination of parameters associated with oxidative stress

The colon tissues were homogenized with tissue lysate buffer containing 1% protease inhibitor and centrifuged at 12,000×g at 4°C for 10 min to obtain the supernatants. The protein concentration was determined using a BCA protein assay kit. T-AOC, SOD, CAT, ROS, and MDA levels in colon tissues were determined using commercial kits according to the manufacturer’s instructions (Nanjing Jiancheng Bioengineering Company). The content or activity of the oxidative parameters was evaluated based on mM/mg protein or U/mg protein.

### Determination of the levels of cytokines and pro-inflammatory mediators

Supernatants obtained from colon tissue were used for ELISA analysis. Levels of TNF-α, IL-6, IL-1β, PGE2, and NO were determined following the manufacturer’s instructions for each assay. Briefly, a 96-well plate coated with capture antibody was blocked with ELISA diluent and incubated for 1 h on an orbital shaker. After washing the plates, the standard sample and experimental samples were added to the designated wells and incubated at 37°C for 2 h with gentle shaking. After incubation, the plate was washed thoroughly and incubated with the primary antibody and subsequently with diluted avidin horseradish peroxidase (Avidin-HRP), washing the plate between each step. Plates were eventually incubated with 1 × 3,3′,5,5′-tetramethylbenzidine (TMB) substrate solution. Subsequently, plates were incubated at room temperature for color development. After adequate color development, the TMB reaction was terminated by adding 50 μL 2 N sulfuric acid (H_2_SO_4_) to each well. The absorbance was measured at 450 nm using a FlexStation^®^ 3 multifunctional microplate reader.

### RT-qPCR

TRIzol^®^ reagent was used to isolate RNA from colon tissues (Invitrogen, Carlsbad, CA, United States). The concentration and purity of the obtained RNA were determined using a NanoDrop™ spectrophotometer (NanoDrop Technologies, Wilmington, DE, United States). Reverse transcription of the purified RNA was performed using a Prime-Script™ RT Reagent Kit (Takara, Japan). Quantitative real-time PCR was conducted using a TB Green™ Premix Ex Taq™ (Tli RNaseH Plus) reagent kit (TaKaRa, Japan), and this reaction was carried out using an ABI 7900HT Real-Time PCR System (Thermo Fisher Scientific, United States). Expression of the target genes was normalized to *β-actin*. The qPCR primer sequences are shown in Supplementary Table S1.

### Western blot

Lysis of the samples was performed in RIPA buffer containing 1 mM PMSF (Beyotime Technology, Shanghai, China) in an ice bath. BCA protein assay kits were used to determine the protein concentration in supernatants (Solarbio, Beijing, China). As in our previous study (X. Wang et al., 2020), an equal amount of protein from each sample was subjected to 10% SDS-PAGE and transferred to PVDF membrane. Primary antibodies (1:500 dilution) were incubated with the membranes at 4 °C overnight. Next, the membranes were incubated with secondary antibodies (1:1000 dilution) at room temperature for 1 h. An ECL kit (Solarbio Co. Ltd., Beijing, China) and a gel documentation system (Gel Doc EZ, BIO-RAD, United States) were used for protein band imaging. Protein band intensities were measured using ImageJ, with levels of *ß*-actin used to normalize the data.

### Gut microbiota analysis

Cecal contents were chosen from each group for microbiota sequencing analysis. Briefly, a QIAamp^®^ Fast DNA Stool MiniKit (Qiagen, Germany) was used to extract total DNA from bacteria according to the manufacturer’s instructions. The DNA content was evaluated using the QuantiT™ dsDNA HS Reagent. Subsequently, an Illumina HiSeq 2500 instrument was used to determine the sequence of the bacterial 16s rDNA. The DNA extraction, quality assessment, and data library construction were carried out by the Microread Genetics Company (Beijing, China). According to the 97% sequence similarity level, the Uclust method in the QIIME software package was used for operational unit (OTU) clustering analysis. Then, based on the Silva database (Release 128 https://www.arb-silva.de/documentation/release-128/), the OTUs of each sample were annotated using Taxonomy. The Ribosomal Database Project was used to analyze the colony structure and beta diversity of the entire sample.

### Statistical analysis

The experimental data obtained in this study are displayed as means ± SD. SPSS^®^ (version 20.0) was used for statistical analysis. One-way analysis of variance (ANOVA) based on least significant difference analysis was used to determine significant differences.

## Results

### Bioactive compound profile of PHE *via* UPLC-LTQ-Orbitrap-MS/MS

**FIGURE 1:**
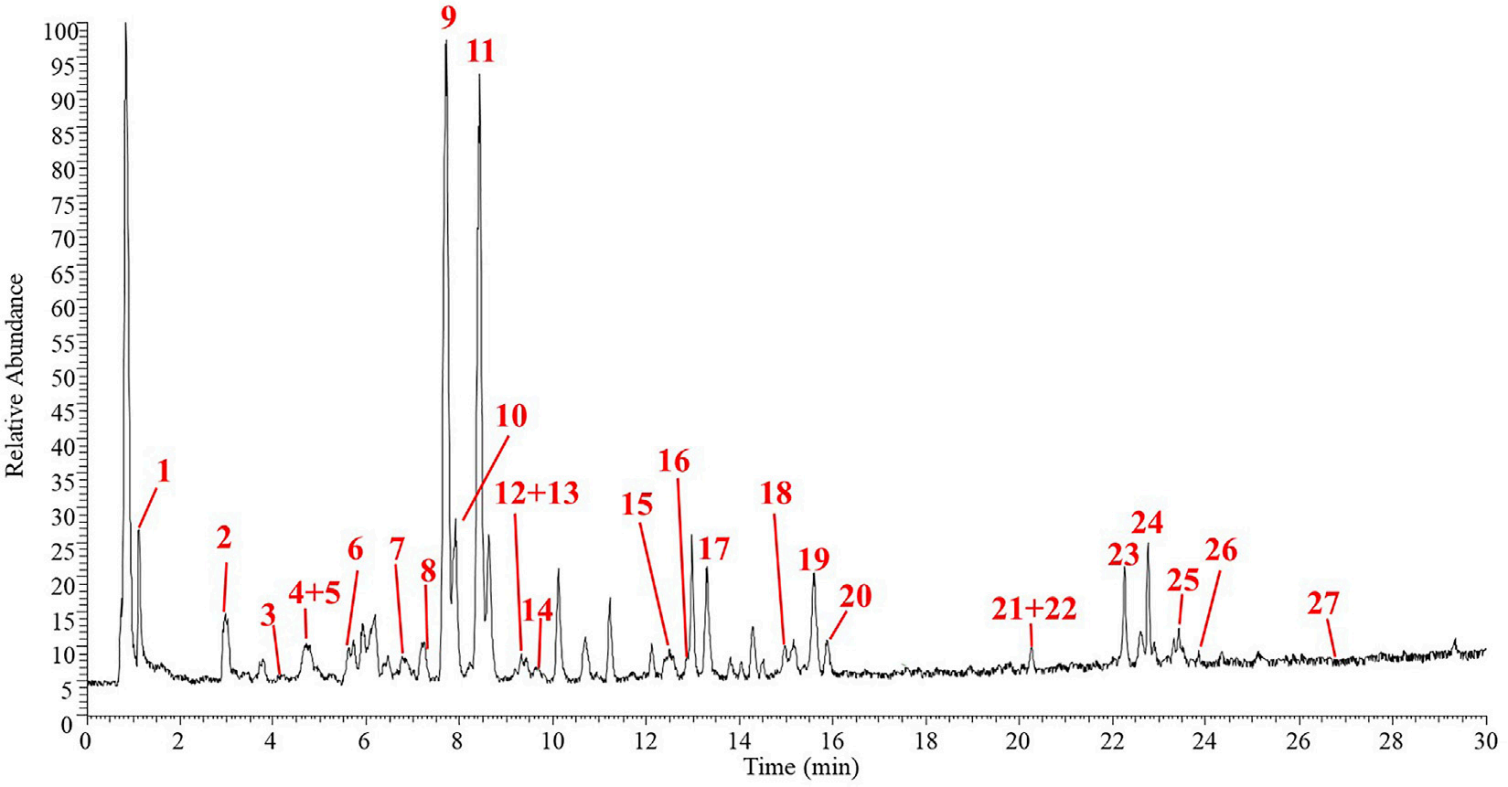
Total ion chromatograms (TIC) of PHE.

The bioactive compound profile of PHE was determined by UPLC-LTQ-Orbitrap-MS/MS through a comparison of the retention times (t_R_), *m/z*, and tandem mass spectrometry (MS^2^) data with data from the literature. A total of 27 compounds were tentatively identified in PHE, including one phenolic acid, 16 flavonoids and glycosides, four isoflavonoids and glycosides, and four saponins, which are summarized in Table 1. Consistent with previous findings, flavonoids, isoflavonoids, and their glycosides were the dominant components of PHE (Lu et al., 2013; Jing et al., 2021). As shown in Figure 1, peak 3 displayed a parent ion [M-H]^-^ at *m/z* 415.1013 that was tentatively identified as puerarin, which is one of the main components of *Flos Puerariae* (Lu et al., 2013). Quercetin, with a parent ion [M-H]^-^ at *m/z* 285.0095, was also found in PHE at peak 7. Peak 8 displayed [M-H]^-^ at *m/z* 607.1630, with fragment ions at *m/z* 313.0043 and 298.0231, and was identified as kakkalide. Peak 11 displayed a mother ion [M-H]^-^ at *m/z* 461.1064 and daughter ions at *m/z* 446.0283 [M-H-CH_3_]^-^, 415.0985 [M-H- C2H_6_O]^-^, and 298.9627[M-H-C_6_H_11_O_5_]^-^, in agreement with prior research (Wei et al., 2022), indicating the presence of tectoridin. For peak 14, the protonated molecule [M-H]^-^ at *m/z* 267.9892, with main fragment ions at *m/z* 267.9892 [M-H-C_6_H_11_O_5_], was detected at 9.59 min in negative ion mode. According to the data, the principal cleavage process maintained deglycosylation, and the fragment ions at *m/z* 267.9892 resulted from the loss of a glucose group. The molecular formula has been estimated as C_21_H_20_O_10_ and the compound was identified as genistin (also called genistein 7-O-glucoside). Peak 16, with a mother ion [M-H]^-^ at *m/z* 285.0394 and a daughter ion at *m/z* 269.0004, suggested the presence of the compound kaempferol. In addition to the substances mentioned previously, detailed information on other identified phenolics and flavonoids is presented in Figure 1 and Table 1. These compounds may contribute to diverse bioactivities, including the antioxidant and anti-inflammatory actions of PHE.

**TABLE 1:** Characterization of the chemical constituents of PHE by UPLC-LTQ-Orbitrap-MS^2^.

No.	t_R_ (min)	[M-H]^-^	MS/MS	Formula	Identification	Source
1	1.11	191.0194	146.8975	C_6_H_8_O_7_	Citric acid	F/S
2	2.97	205.0711	186.9879, 143.0246, 115.0560	C_11_H_12_N_2_O_2_	Tryptophan	F/S
3	4.41	415.1013	--	C_21_H_20_O_9_	Puerarin	F
4	4.63	609.1431	563.0222, 428.0886	C_27_H_30_O_16_	Rutin	F
5	4.69	353.1495	191.0101, 172.9663, 110.9995	C_16_H_18_O_9_	4-Caffeoylquinic acid	F
6	5.60	623.1586	461.0663	C_28_H_32_O_16_	6-Hydroxychickpea sprout a-6,7-diglucoside	F/S
7	6.84	301.0706	285.0095	C_15_H_10_O_7_	Quercetin	F/S
8	7.41	607.1630	313.0043, 298.0231	C_28_H_32_O_15_	Kakkalide	F
9	7.69	593.1473	473.0505, 413.1399	C_27_H_30_O_15_	Vitexin 2″-O-β-d-glucopyranoside/isovitexin 2″-O-β-d-glucopyranoside	F/S
10	8.28	577.1524	431.0248, 298.9164, 284.9632	C_27_H_30_O_14_	Puerarin-4′-O-β-d-glucopyranoside	F
11	8.41	461.1064	446.1283, 415.0985, 340.9573, 298.9627	C_22_H_22_O_11_	Tectoridin	F
12	9.31	623.1939	461.0663	C_28_H_32_O_16_	6-Hydroxybiochanin A-6,7-di-O-glucoside	F
13	9.41	447.0909	284.9692	C_21_H_20_O_11_	6-Hydroxygenistein-7-oglucoside	F
14	9.59	431.0965	267.9892	C_21_H_20_O_10_	Genistin	F
15	12.57	283.0601	268.0292, 254.9701, 238.9880	C_16_H_12_O_5_	Kakkatin	F
16	12.89	285.0394	269.0004, 257.0361, 240.9996	C_15_H_10_O_6_	Kaempferol	F/S
17	13.30	463.1425	447.0141, 299.9738	C_22_H_24_O_11_	Dihydrotectoridin	F
18	15.14	269.0448	240.0180, 225.0333, 201.0156	C_15_H_10_O_5_	Genistein	F
19	15.61	299.0548	283.9724, 255.0026	C_16_H_12_O_6_	Tectorigenin	F
20	15.89	475.1213	355.0140, 313.0153	C_23_H_24_O_11_	Kakkalidone	F
21	20.25	287.2219	269.1670, 241.1514, 214.9835	C_15_H_12_O_6_	Dihydrokaempferol	S
22	20.68	331.1900	--	C_16_H_12_O_8_	Laricitrin	F/S
23	22.23	941.5056	923.3801, 779.3140, 509.2721, 439.2111	C_48_H_78_O_18_	Soyasaponin I	F
24	22.75	925.5101	907.4330, 863.4353, 779.3845, 717.4513	C_48_H_78_O_17_	Kaikasaponin III	F
25	23.42	895.5012	779.4547, 549.1013, 311.2217, 161.0454	C_47_H_76_O_16_	Kakkasaponin I	F
26	23.83	923.4968	905.4011, 861.4161, 715.4210, 597.3115	C_48_H_76_O_17_	Phaseoside IV	F
27	26.94	317.2110	248.9568, 180.9362	C_15_H_10_O_8_	Myricetin	S

F: *Flos Puerariae*; S: *Semen Hoveniae*.

### Impact of PHE on body weight, DAI score, and colon length

**FIGURE 2:**
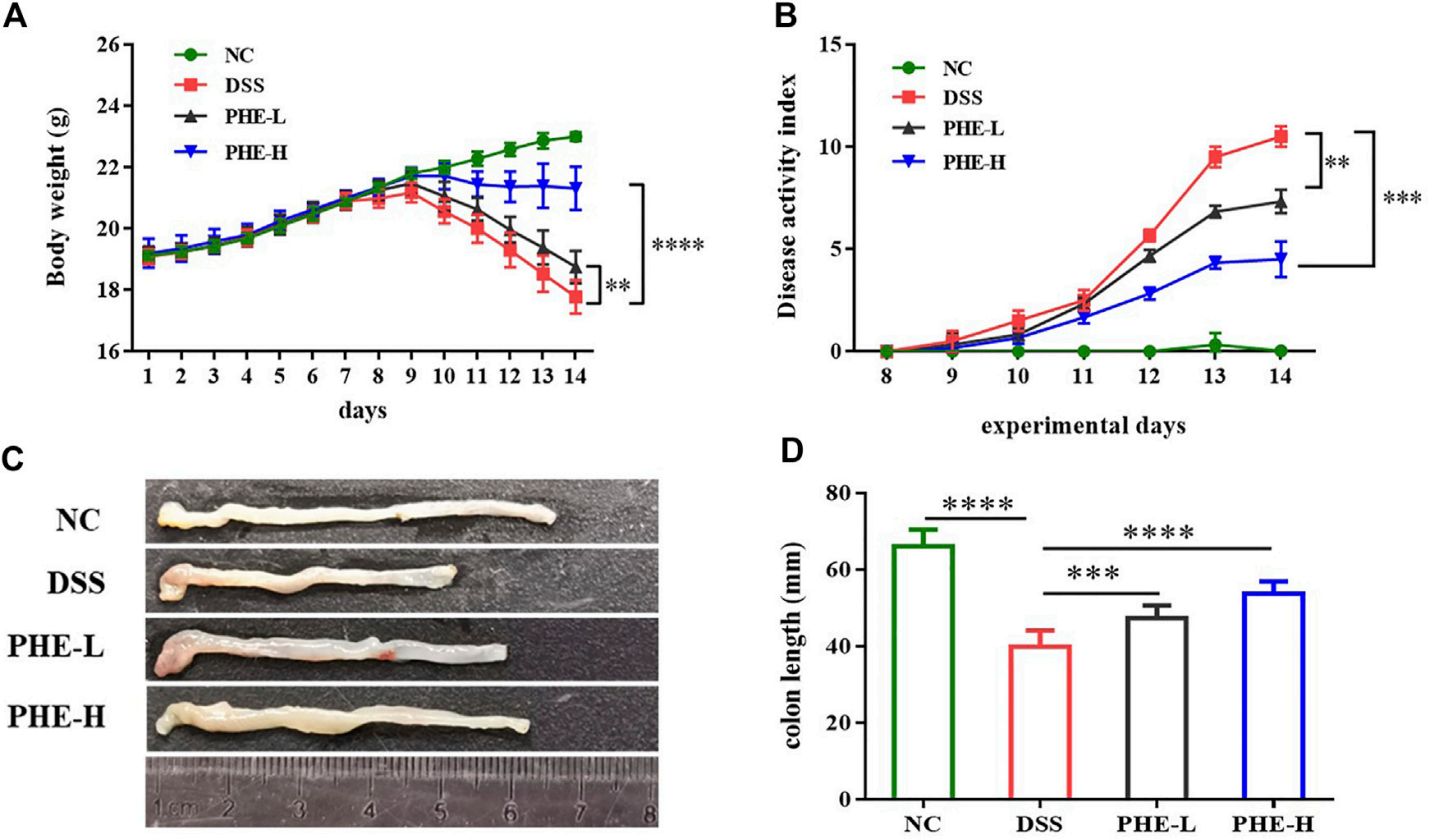
Effect of PHE supplementation on colitis mouse. Body weight **(A)**. DAI score **(B)**. representative images **(C)** and data of the colon length **(D)**. Graphs show mean ± SD; *n* = 8; **p < 0.01, ***p < 0.001, and ****p < 0.0001.

Subsequently, a DSS-induced colitis mouse model was used to explore the efficacy of PHE in modulating inflammatory responses. Body weights of the mice were recorded throughout the experiment (Figure 2A). The DSS-treated groups exhibited a significant reduction in body weight from day 8 until the last day of the experiment, and PHE treatment significantly inhibited body weight loss, even in the low-dose group (*p* < 0.01). Consistent with the change in body weight, the DAI in the DSS-treated group exhibited the highest value among all treatment groups, likely due to DSS-induced weight loss, bloody stools, and rectal bleeding (Figure 2B). PHE administration effectively attenuated the DAI in both the low-dose (*p* < 0.01) and the high-dose (*p* < 0.001) PHE intervention groups. Reduction in colon length is a characteristic of colitis; therefore, we measured colon length after treatment. Colon length in the DSS-treated group decreased to nearly half of that of the NC group (*p* < 0.0001), whereas PHE administration markedly attenuated the reduction in colon length (Figures 2C,D, *p* < 0.001) in a concentration-dependent manner. The aforementioned data indicate that DSS induced a reduction in body weight and colon length and led to bloody stools, whereas PHE administration significantly attenuated these effects of colitis.

### Protective effects of PHE against colon damage in mice with colitis

**FIGURE 3:**
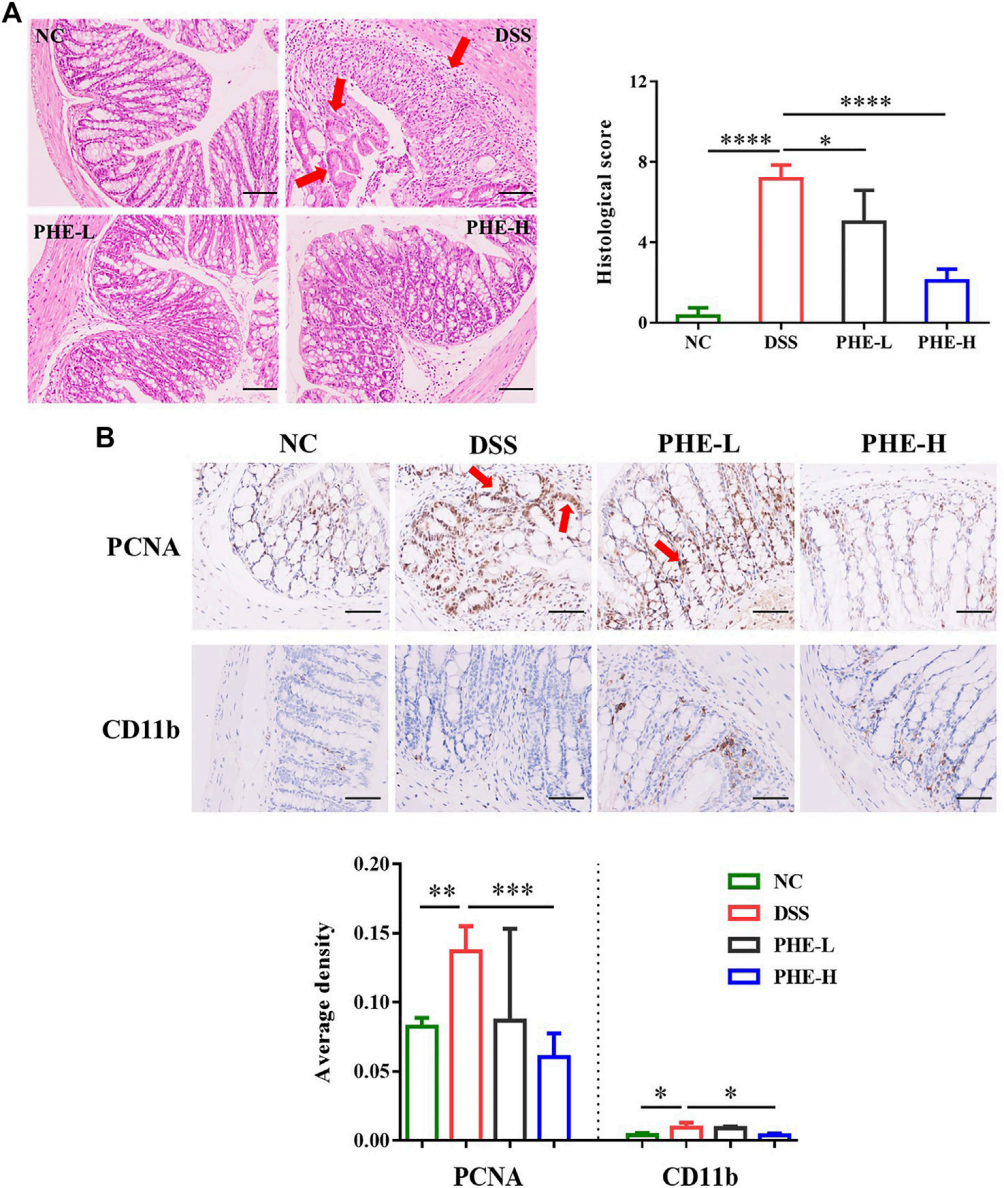
Representative H&E-stained **(A)** distal colon sections (scale bar 100 µm); PCNA and CD11b expression **(B)** levels in colon tissue (scale bar 50 µm); the magnification factor is 200 and 400×, respectively. The results are expressed as mean ± SD; *n* = 4; *p < 0.05, **p < 0.01, ***p < 0.001, and ****p < 0.0001.

To further determine the ameliorating effects of PHE supplementation in the context of colon injury, inflammatory damage in the colon tissues was assessed using H&E staining and immunohistochemistry analysis. As shown in Figure 3A, compared with the NC group, DSS induced severe crypt distortion, epithelial damage, mucosal edema, and immune cell infiltration in the mouse colon. Furthermore, in the DSS group, the H&E score was significantly higher than that of the NC group (*p* < 0.0001), whereas both low- and high-dose PHE treatment significantly mitigated the morphological changes caused by DSS in the colon by restoring epithelial structure and reducing immune cell infiltration. PCNA and CD11b expression was evaluated in immune cells to assess cell proliferation and adhesive interactions. As demonstrated by immunohistochemical analysis, the number of PCNA and CD11b positive cells was significantly increased in the DSS group, whereas supplementation with high-dose PHE effectively reduced the number of PCNA and CD11b positive cells (*p* < 0.05, Figure 3B); effects with low-dose PHE were not significantly different from those observed in the DSS group (*p* > 0.05).

### Effect of PHE treatment on oxidative stress in mice with colitis

**FIGURE 4:**
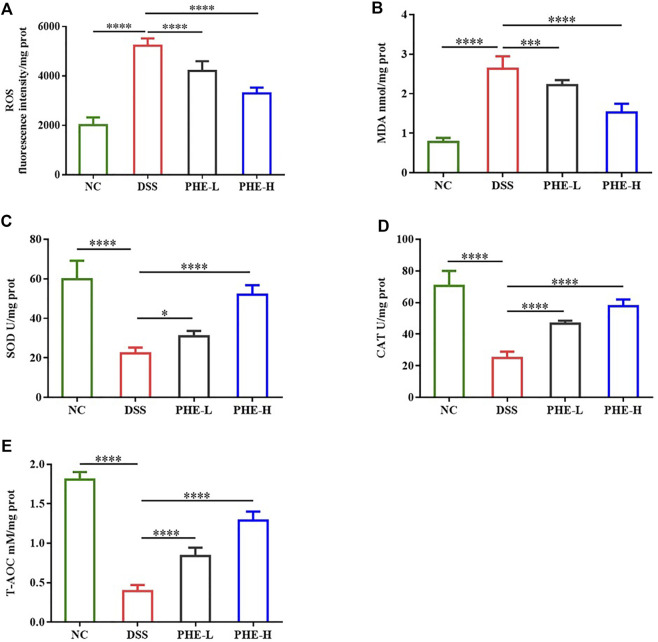
Effect of PHE administration on colonic oxidative stress parameters in the DSS-induced colitis mouse model. **(A)** ROS; **(B)** MDA; **(C)** SOD; **(D)** CAT; **(E)** T-AOC. The results are expressed as mean ± SD; *n* = 8; *p < 0.05, **p < 0.01, ***p < 0.001, and ****p < 0.0001.

As oxidative stress is considered a critical factor in gut inflammation, oxidative stress in DSS-induced mice was assessed. The levels of ROS and MDA in the DSS group were more than double those in the NC group (*p* < 0.0001), indicating severe oxidative stress in colon tissues induced by DSS. In contrast, PHE treatment markedly attenuated the levels of ROS and MDA at both low and high doses (*p* < 0.001, Figures 4A,B). Furthermore, evaluation of the activities of SOD and CAT demonstrated that DSS treatment significantly reduced their activities, while PHE administration restored these antioxidant enzyme activities (*p* < 0.05, Figures 4C,D). Consistent with this, the T-AOC was also improved by PHE treatment in a dose-dependent manner (*p* < 0.0001, Figure 4E). These data indicate that PHE significantly reduced oxidative stress in colon tissue, which suggests a mechanism of attenuation of colitis-associated damage in the gut.

### Effect of PHE on the levels of inflammatory cytokines in colon tissue

**FIGURE 5:**
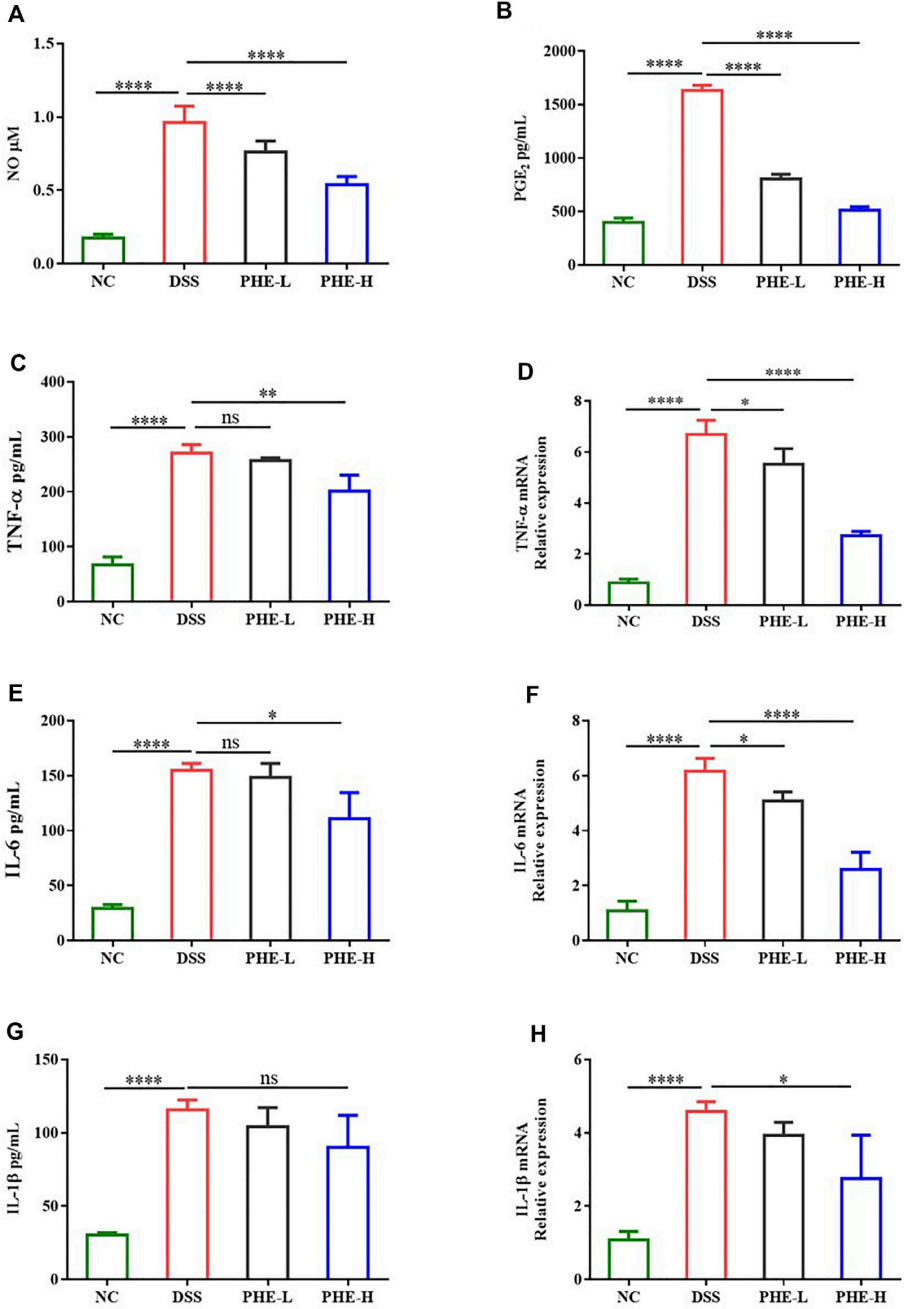
Effect of PHE on the level of **(A)** NO, **(B)** PGE2, **(C)** TNF-α, **(E)** IL-6, and **(G)** IL-1β and the corresponding expression of **(D)** TNF-α, **(F)** IL-6, and **(H)** IL-1β mRNA in colon tissues. The results are expressed as mean ± SD; *n* = 8; *p < 0.05, **p < 0.01, ***p < 0.001, and ****p < 0.0001.

To further explore whether PHE modulates the intestinal immune response, the levels of IL-6, TNF-α, IL-1β, NO, and PGE2 were determined using ELISA and biochemical assay kits. As displayed in Figures 5A,B, DSS treatment greatly increased the production of NO and PGE2 relative to that in the NC group, while PHE administration reduced the inflammatory response, even in the group treated with a low dose of PHE (*p* < 0.0001). Furthermore, the ELISA results indicate that the expression levels of TNF-α and IL-6 were significantly elevated by DSS treatment (Figures 5C,E, *p* < 0.05), but were suppressed by high-dose PHE. The expression of IL-1β was not significantly different when comparing the DSS and high-dose PHE treatment groups (*p* < 0.05, Figure 5G). We further determined the expression levels of the corresponding mRNA. The abundance of these pro-inflammatory genes was significantly higher in DSS-induced mice (*p* < 0.0001) and expression was suppressed by PHE treatments (*p* < 0.05, Figures 5D,F,H). All these data suggest that PHE, especially at the high dose, effectively reduced the production of pro-inflammatory cytokines in the DSS-induced mouse model, leading to the preservation of mucosal immune homeostasis in the colon.

### Effect of PHE treatment on the activation of the iNOS, COX-2, and MAPK pathways in mice with colitis

**FIGURE 6:**
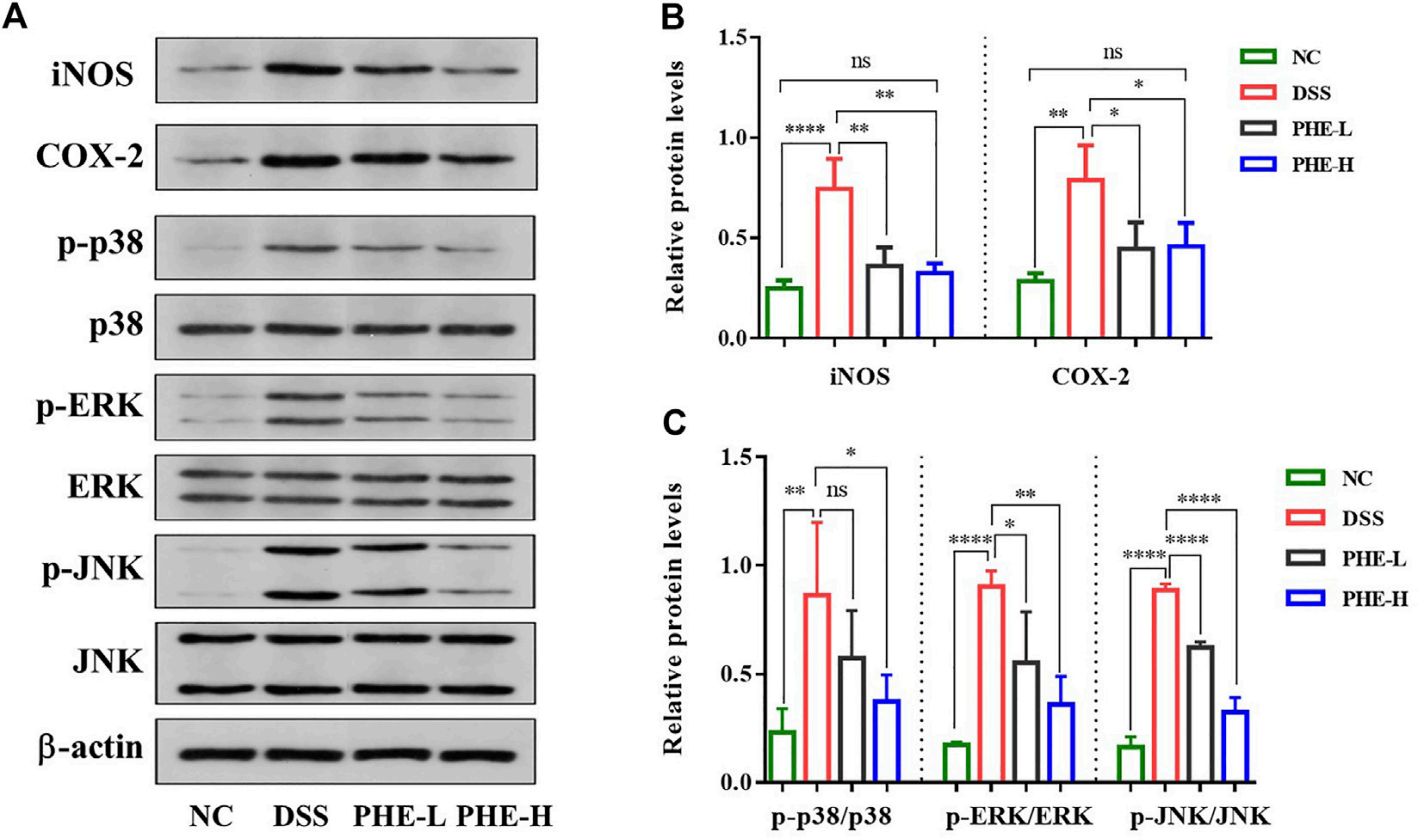
Effect of PHE on iNOS, COX-2 and MAPK signaling pathway in colon tissues. Characteristic picture of western blot **(A)**, expression level of iNOS, COX-2 **(B)**, and MAPK **(C)** signal pathway. The results are expressed as mean ± SD; *n* = 8; *p < 0.05, **p < 0.01, ***p < 0.001, and ****p < 0.0001.

To determine how PHE modulates DSS-induced colitis, we evaluated the expression levels of iNOS, COX-2, and other proteins in the MAPK signaling pathway. Western blot results showed that the expression of iNOS and COX-2 was obviously increased in the DSS group relative to the NC group, but was inhibited by PHE at both high and low doses (*p* < 0.05, Figures 6A,B). Additionally, the expression levels of p-ERK, p-p38, and p-JNK were significantly elevated in the DSS group (Figures 6A,C). Except for the expression of p-p38, both low- and high-dose PHE markedly inhibited the expression of proteins of the MAPK signaling pathway. These results demonstrate that PHE treatment may attenuate the secretion of cytokines and mediators by blocking the activation of iNOS, COX-2, and the MAPK signaling pathway.

### Effect of PHE on composition of the gut microbiota

**FIGURE 7:**
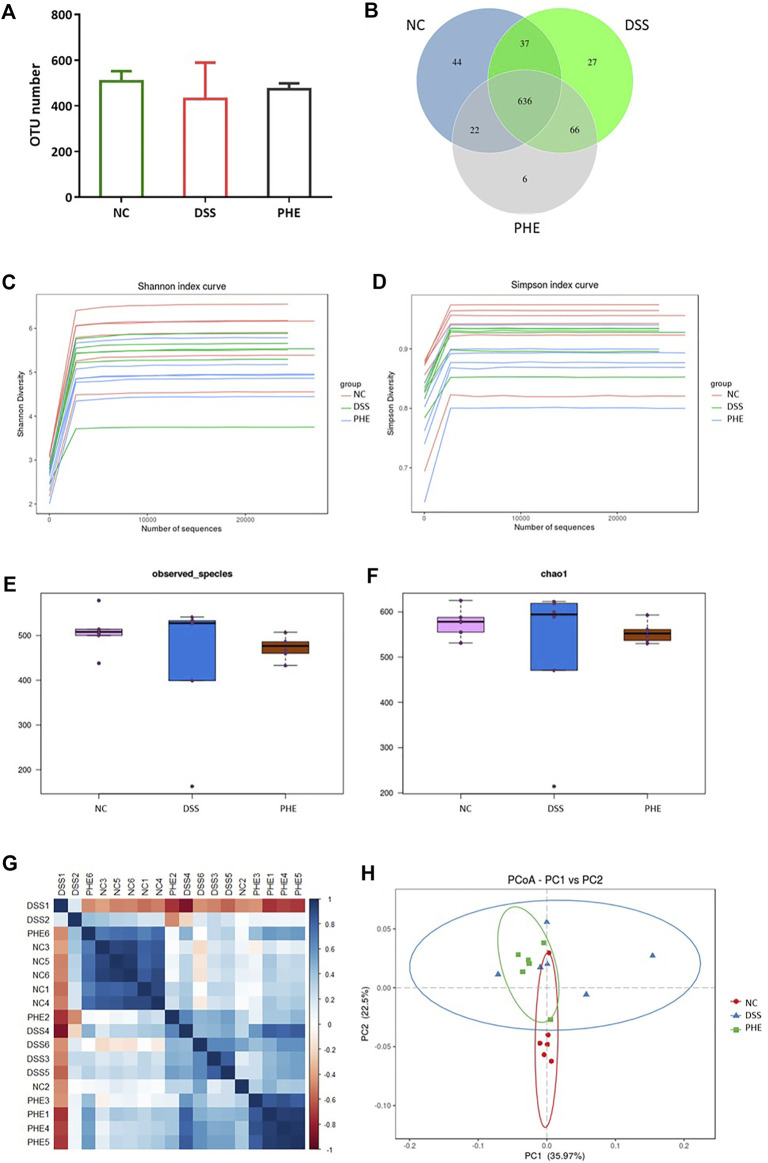
Effect of PHE on composition of the gut microbiota. **(A)** OTU numbers in different groups. **(B)** OTU Venn diagram. **(C)** Shannon index curve. **(D)** Simpson index curve. **(E)** Observed_species index. **(F)** Chao1 index. **(G)** Distance matrix heat map. **(H)** PCoA; *n* = 6.

Animal models of IBD have revealed dysbiosis of the gut microbiota and emphasize its vital role in the disease process (Shah and Itzkowitz, 2022). Based on 16s RNA sequencing of cecal contents from mice in the NC, DSS, and high-dose PHE groups, we explored whether PHE supplementation protected mice from DSS-induced IBD by alteration of the microbiome structure and composition. As shown in Figure 7A, 1429 OTUs were detected across these groups. DSS treatment reduced the OTU number compared with the NC group, whereas PHE pretreatment inhibited the reduction in OTU number, resulting in no significant difference from the NC group (*p* > 0.05). There were 636 OTUs detected in the three groups, whereas six OTUs were detected in the PHE group that were different from those of the NC and DSS groups (Figure 7B). Alpha diversity, including Shannon, Simpson, observed_species, and Chao1, estimates were used to display the richness and diversity of the species. As shown in Figures 7C,D, the Shannon and Simpson curves rose sharply with the increase in the number of sequences and then approached equilibrium, demonstrating that the number of sequences obtained from the current samples was sufficient and reasonable. The observed_species and Chao1 indices in Figures 7E,F indicate that the diversity in the DSS group was reduced relative to the NC group, while PHE treatment increased the diversity of the species. The similarity of species among the three groups was further analyzed by beta diversity using principal coordinates analysis (PCoA). Figure 7G displays the unweighted UniFrac distance matrix heat map for the NC, DSS, and PHE groups, indicating that the species composition of the NC was different from that of the DSS and PHE groups. Based on the unweighted UniFrac distance matrix, PcoA analysis also suggested a different species composition when comparing the DSS and PHE groups (Figure 7H).

**FIGURE 8:**
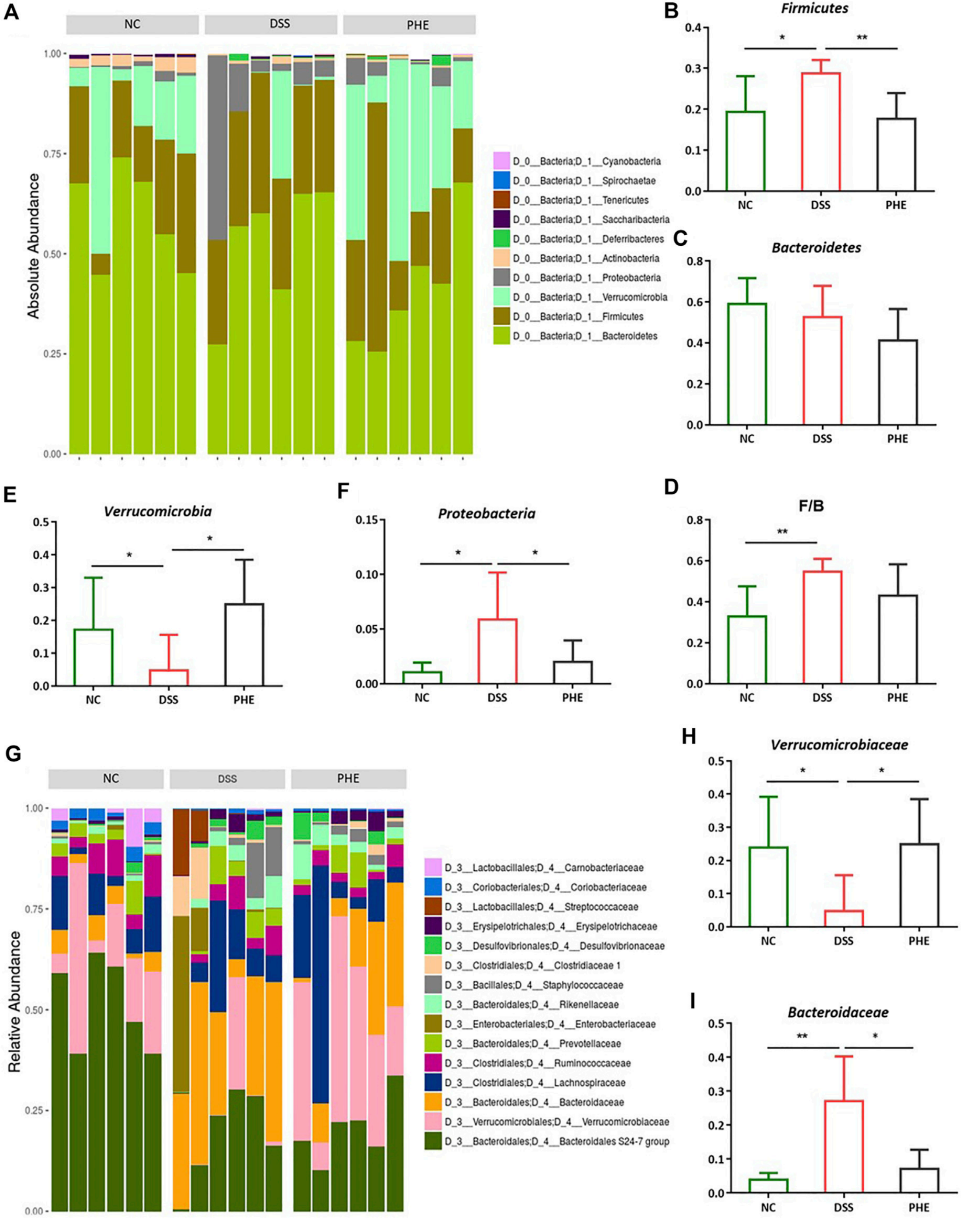
Effect of PHE on gut microbiota in phylum and family level; Gut microbiota at the phylum levels **(A)**, the level of Firmicutes **(B)**, Bacteroidetes **(C)**, F/B ratio **(D)**, Verrucomicrobia **(E)**, Proteobacteria **(F)**; Gut microbiota at the family levels **(G)**, the level of Verrucomicrobiaceae **(H)**, Bacteroidaceae **(I)**. n = 6. * p < 0.05, ** p < 0.01, *** p < 0.001 and **** p < 0.0001.

**FIGURE 9:**
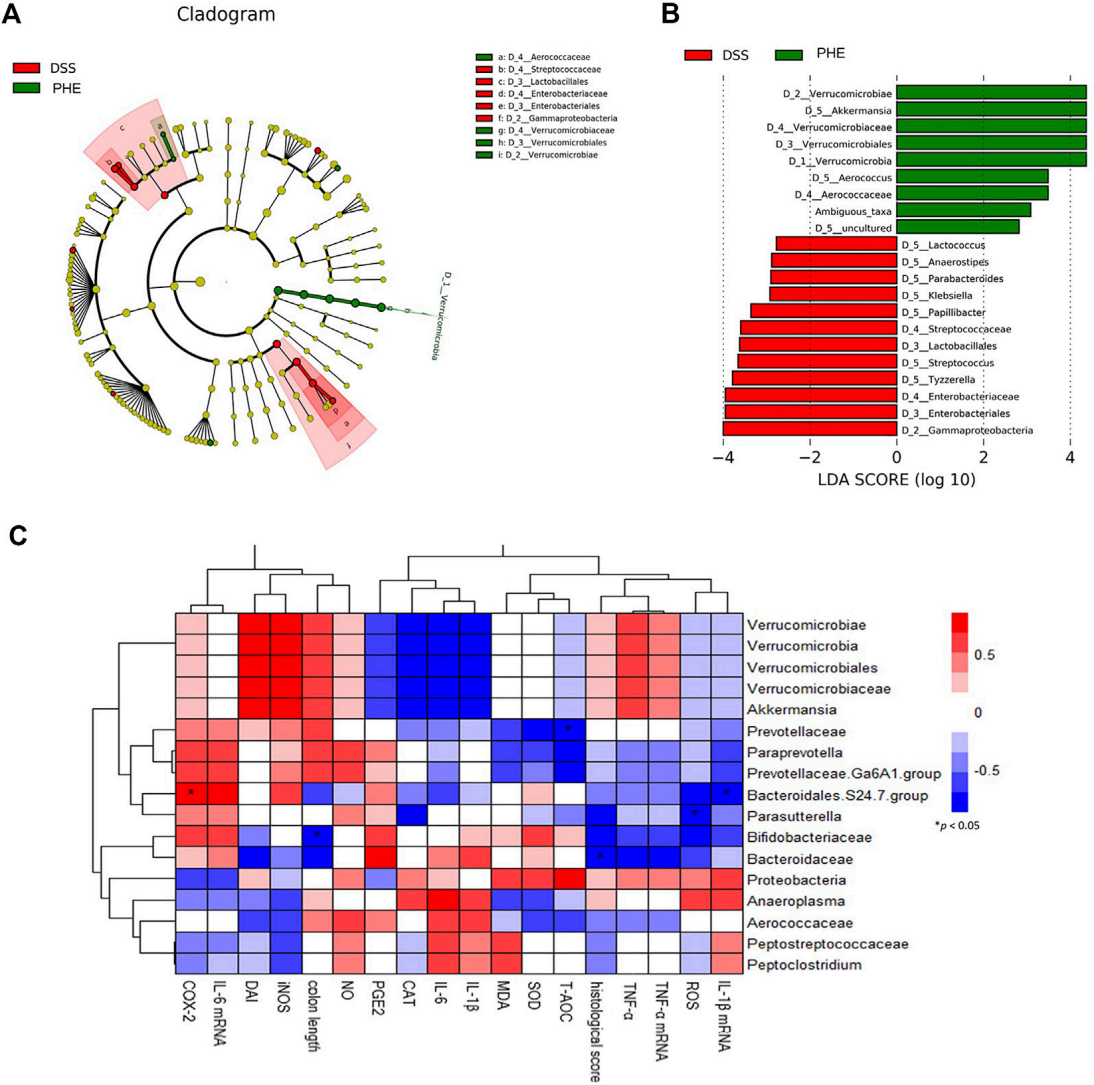
Significant differences in microbiota of the DSS and PHE treatment groups *via* LEfSe analysis. **(A)** Evolutionary branching diagram. **(B)** Taxa meeting an LDA score threshold >2.5. **(C)** Correlation between key communities of gut microbiota and parameters of colitis in PHE-treated mice. *Spearman’s correlation indicates significant difference at a level of 0.05; *n* = 6.

To confirm which species were regulated by PHE, the species compositions at the phylum and family levels were determined. At the phylum level, the gut microbiota of the NC, DSS, and PHE groups consisted mainly of *Bacteroidetes*, *Firmicutes*, and *Verrucomicrobia* (Figure 8A). There was a substantial increase in the abundance of *Firmicutes* in the DSS mice relative to the NC group, while the abundance of *Verrucomicrobia* decreased (both *p* < 0.05, Figures 8B,E). *Bacteroidetes* decreased in both the DSS and PHE groups, but the difference was not significant relative to the NC group (*p* > 0.05, Figure 8C). Consistent with this, the *Firmicutes/Bacteroidetes* ratio was elevated in the DSS group due to an increased abundance of *Firmicutes*, indicating severe inflammation in the colon (Figure 8D). *Proteobacteria* were abundant in the DSS group but were suppressed by PHE treatment (*p* < 0.05, Figure 8F). A previous study reported a positive relationship between *Proteobacteria* and intestinal dysfunction (Chopyk and Grakoui, 2020). At the family level (Figure 8G), the relative abundance of *Verrucomicrobiaceae* was significantly reduced in the DSS group relative to that in the NC group, whereas the relative abundance of *Bacteroidaceae* was significantly enhanced (both *p* < 0.05, Figures 8H,I). PHE treatment significantly increased the abundance of *Verrucomicrobiaceae* and reduced the abundance of *Bacteroidaceae*. A linear discriminant analysis effect size (LefSe) analysis was constructed, and species with an LDA score of greater than 2.5 were identified as significant biomarkers. As shown in Figure 9B, *Verrucomicrobiae, Akkermansia, Verrucomicrobiaceae, Verrucomicrobiales, Verrucomicrobia*, and *Aerococcus* were the dominant species in the PHE group, whereas *Gammaproteobacteria*, *Enterobacteriaies*, and *Enterobacteriaceae*, among others, were dominant in the DSS group. The evolutionary branching diagram of the LEfSe analysis (Figure 9A) also indicated different biomarkers in the DSS and PHE groups. At the end of the survey, a correlation heat map was used to calculate the correlation between the inflammatory markers, including colon length, cytokines, oxidative parameters, and gut microbiota. The results suggest that these florae were either positively or negatively correlated with oxidative and inflammatory markers. For example, *Bacteroidaceae* and *Prevotellaceae* were significantly negatively correlated with histological score (*p* < 0.05), whereas the *Bacteroidales.S24.7.group* was significantly correlated with COX-2 expression (Figure 9C). These findings indicate that supplementation with PHE can alter the abundance and composition of the gut microbiota by enhancing beneficial bacteria, such as *Verrucomicrobia* (Fujio-Vejar et al., 2017), and reducing harmful bacteria, such as *Proteobacteria*.

## Discussion

A growing number of research studies have highlighted the complex effects of plant-derived bioactive compounds on human health, and flavonoids and other phytochemicals have undergone extensive investigation. The primary bioactive constituents of *Flos Puerariae* and *Semen Hoveniae* have been identified as flavonoids, saponins, volatile oils, alkaloids, sterols, and amino acids (Wei et al., 2022). In the present study, the bioactive profile of extract from the *Flos Puerariae* and *Semen Hoveniae* medicinal pair was evaluated using UPLC-LTQ-Orbitrap-MS/MS. Consistent with previous reports, flavonoids, isoflavonoids, and their glycoside derivatives (such as puerarin, rutin, quercetin, vitexin 2″-O-β-d-glucopyranoside, tectoridin, kakkatin, and kaempferol) accounted for most of the bioactive compounds in PHE (Table 1). Similarly, Wei et al. investigated the chemical constitution of *Flos Puerariae* and *Semen Hoveniae* extracts by HPLC and Fourier-transform ion cyclotron resonance mass spectrometry and identified 48 chemical constituents, including genistin, tectoridin, glycitein, genistein, and tectorigenin (Wei et al., 2022). These bioactive substances derived from *Flos Puerariae* and *Semen Hoveniae* may have therapeutic efficacy in inflammation-related diseases. For example, puerarin, one of the main flavonoids found in PHE, can attenuate lipopolysaccharide (LPS)-induced inflammatory injury in gastric epithelial cells by repressing the AMPK/SIRT1/NLRP3 signaling pathway (Peng and Liu, 2022). Kakkalide, separated from *Pueraria lobata*, significantly inhibited LPS-stimulated NF-κB activation and TNF-α expression in macrophages and suppressed TNBS-induced colitis in mice (Jang et al., 2019). The anti-inflammatory property of kaempferol, a flavonoid found in a variety of dietary sources, appears to be related to its reduction of activation of TLR4/NF-κB signaling (Bian et al., 2022). The potential beneficial effects, including antioxidant, hepatoprotective, anti-cancer, and cardioprotective effects, of genistin, the main isoflavone glycoside identified in PHE, have been reviewed (Islam et al., 2020) Genistin displays effective antioxidant properties through scavenging and reducing the activities of free radicals (B.-S. Wang et al., 2012). Oxidative stress and related inflammatory responses are closely associated with a variety of acute and chronic diseases, including intestinal colitis and liver injury. Therefore, the bioactive compounds isolated from PHE provide a reliable foundation for their physiological activities.

The intestinal barrier is a complex system that consists of a mucus layer, epithelial cells, and immune cells that harbor commensal bacteria and protect the host against various pathogens, such as harmful bacteria and food antigens. Dysfunction of the intestinal barrier is involved in the pathogenesis of various diseases, including IBD (Chopyk and Grakoui, 2020). Shortened colon length, increased DAI, and damaged colon tissue are the characteristic symptoms of colitis. The results of our study indicate that oral administration of PHE ameliorates colitis by restoring colon length, reducing DAI, and decreasing pathological injury of the colon. Immunohistochemical analysis indicated that PHE inhibits inflammatory cell infiltration in colonic tissues and restores intestinal epithelial structure. It has been reported that damaged intestines are more vulnerable to external stimulation, such as physical injury, chemical stress, or redox imbalance (Martel et al., 2022). As previously reported, inflammation is aggravated by the release of reactive oxygen species (ROS). Elevated ROS levels result in oxidative stress and tissue damage (Zhang and Tsao, 2016). In addition, ROS-mediated mechanisms such as ERK signaling result in increased levels of pro-inflammatory cytokines, which lead to chronic inflammation (Ziauddeen et al., 2019).

Phytochemicals are powerful natural antioxidants due to their capacity to scavenge free radicals. Phytochemicals protect human tissues from oxidative damage and the resulting effects. A previous report demonstrated that polyphenol-rich *Forsythia suspensa* extract relieved the symptoms of colitis by reducing the level of ROS through modulation of the Nrf2-NLRP3 pathway (Chao et al., 2022). Researchers have investigated the protective effect of extracts rich in quercetin and kaempferol from green pea (*Pisum sativum L*.) hulls and found that the extracts displayed significant antioxidant capacity by reducing MDA content and enhancing the activity of SOD and CAT in mice with DSS-induced colitis (Guo et al., 2021). Caffeic acid has also exerted protective effects against colonic inflammation by increasing the levels of GSH-Px, CAT, and SOD in DSS-induced colitis (Wan et al., 2021). Likewise, our study demonstrated that PHE, which contains various phenolic acids and flavonoids, displayed excellent antioxidant properties by reducing ROS and MDA, increasing the activity of SOD and CAT, and ultimately enhancing T-AOC. These results demonstrate that PHE exerts anti-inflammatory effects by ameliorating oxidative damage in the colon.

Furthermore, it has been demonstrated that inflammatory mediators, such as interleukins (IL-6 and IL-1β), TNF-α, and PGE2, and inflammatory enzymes, such as iNOS and COX-2, are secreted by activated monocytes and macrophages when on-site oxidative stress and inflammation occurs (Ambriz-Pérez et al., 2016). However, the overproduction of cytokines may lead to chronic or severe tissue damage in the colon. Many studies have shown that natural compounds, including phenolic acids and flavonoids, inhibit the secretion of pro-inflammatory mediators, eventually resulting in anti-inflammatory activity (Ambriz-Pérez et al., 2016). For instance, quercetin has been reported to inhibit the production of COX and block pro-inflammatory cytokines by inhibiting MAPK, thus exerting anti-inflammatory action *in vivo* (Ziauddeen et al., 2019). Several researchers have demonstrated that dietary genistein eases colonic injury and downregulates the expression of cytokines by repressing TLR4/NF-κB signaling (R. Zhang et al., 2017). The results obtained in this study indicate that high-dose PHE treatment significantly reduced the production of NO, PGE2, TNF-α, and IL-6. Furthermore, the mRNA expression levels of TNF-α, IL-6, and IL-1β were reduced by PHE. To investigate the mechanism underlying the inhibitory effects of PHE on pro-inflammatory events, we evaluated signal transduction by the MAPK pathway. Our results revealed that PHE treatment significantly inhibited the phosphorylation of p38, ERK, and JNK. MAPK signaling is believed to be involved in the regulation of pro-inflammatory cytokine production and the upregulation of pro-inflammatory enzymes, such as COX-2 and iNOS, in intestinal epithelial cells (Plastira et al., 2020). Our results demonstrate that PHE administration regulated DSS-induced colitis in mice by inhibiting the phosphorylation of proteins in the MAPK signaling pathway.

Currently, intestinal bacteria are considered one of the key elements contributing to the modulation of host health. Intestinal flora imbalance has been associated with several intestinal and extraintestinal disorders, such as IBD, allergic diseases, celiac disease, and chronic liver disease (Cani, 2018; Z. Wang et al., 2021). In the present study, we found differences in the microbiota signature of mice in the NC group compared to those with DSS-induced colitis. DSS treatment induced a decrease in microbial *a* and *ß* diversity, as indicated by observed_species, the Chao1 index, and PCoA analysis, which is often observed in IBD mouse models and patients. The steady state and equilibrium of the microbiota can be evaluated based on its diversity (Fassarella et al., 2021). In line with the change in microbial diversity, a significant increase in *Firmicutes* and *Proteobacteria* was observed in the colon of mice with colitis. Increased proportions of *Proteobacteria* have been reported to be closely associated with inflammation and disease (Hiippala et al., 2018). This may be attributed to the production of a hexa-acylated form of LPS by *Proteobacteria* that causes intestinal inflammation (Di Lorenzo et al., 2019). In addition, a reduction in the abundance of *Verrucomicrobia* was observed in the DSS-treated group, whereas PHE treatment restored its abundance. LEfSe analysis demonstrated that *Akkermansia* was significantly increased in the PHE-treated group. The beneficial effect of *Akkermansia* has attracted wide interest, as it is the only cultivated intestinal representative of *Verrucomicrobia*. Notably, researchers have observed lower levels of *Akkermansia* in patients with inflammatory bowel disease and metabolic disorders, suggesting that it may be useful in such cases (Derrien et al., 2017). *Akkermansia muciniphila* (*A. muciniphila*) is a common commensal bacterium isolated from human feces (Ghaffari et al., 2022). It is a Gram-negative, aerobic, non-motile, oval-shaped, mucin-degrading bacterium found in the mucus layer of the intestinal epithelium. This indicates that PHE can sustain the redox balance in the gut, leading to improved gut barrier function, which is beneficial for the growth of *A. muciniphila*. Numerous studies have shown that *A. muciniphila* can regulate the host immune system *via* necrotizing TNF-α, interferon-gamma (INF-γ), and interleukin-10 (IL-10) (Derrien et al., 2011). The relationship between variables related to colitis and the core microbiota communities in the PHE group demonstrated that the abundance of *Akkermansia* was negatively related to the expression of PGE2, IL-6, and IL-1β. According to these findings, PHE may alleviate colitis by selectively increasing the abundance of benign bacteria, such as *Akkermansia*, and reducing the growth of harmful bacteria, such as *Proteobacteria*.

In summary, the bioactive compounds contained in the extract of *Flos Puerariae–Semen Hoveniae*, particularly phenolic acids and flavonoids, such as puerarin, quercetin, genistin, and kakkalide, conferred effective intestinal protection from DSS-induced colitis in mice through multiple mechanisms. Briefly, PHE treatment 1) significantly ameliorated physiologic damage; 2) reduced ROS production and improved antioxidant enzyme activity; 3) inhibited the phosphorylation of proteins in the MAPK signaling pathway; 4) decreased the expression of cytokines, including TNF-α, IL-6, and IL-1β, and corresponding mRNA levels to modulate oxidative stress in the colon; and 5) alleviated the dysbiosis of intestinal flora. These findings suggest that PHE is protective against DSS-induced intestinal dysfunction by restoring redox balance and the structure of the gut microbiota. It is vital to understand the role of PHE in modulating gut health and in treating related intestinal diseases, including IBD.

## Data Availability

The datasets presented in this study can be found in online repositories. The names of the repository/repositories and accession number(s) can be found in the article/Supplementary Material.
