# Supplementary material for: Flos Puerariae-Semen Hoveniae medicinal pair extract ameliorates DSS-induced inflammatory bowel disease through regulating MAPK signaling and modulating gut microbiota composition
Source: Front Pharmacol. 2022 Dec 7;13:1034031. doi: 10.3389/fphar.2022.1034031 (PMC9768334; doi:10.3389/fphar.2022.1034031)
Supplement: Supplementary file 1 [file Table1.DOCX]

|  | Primer | Sequence | Size |
| --- | --- | --- | --- |
| mus β-actin | Forward | 5‘- CACGATGGAGGGGCCGGACTCATC -3’ | 240bp |
|  | Reverse | 5‘- TAAAGACCTCTATGCCAACACAGT -3’ |  |
| mus TNF-α | Forward | 5‘-AGCACAGAAAGCATGATCCG-3’ | 212bp |
|  | Reverse | 5‘-CTGATGAGAGGGAGGCCATT-3’ |  |
| mus IL-1β | Forward | 5‘-TCAGGCAGGCAGTATCACTC-3’ | 250bp |
|  | Reverse | 5‘-AGCTCATATGGGTCCGACAG-3’ |  |
| mus IL-6 | Forward | 5‘-CACAGAGGATACCACTCCCAACAGA-3’ | 124bp |
|  | Reverse | 5‘-ACAATCAGAATTGCCATTGCACAAC-3’ |  |

Supplementary table 1
